# Safe performance of spinal anesthesia in a critical patient with neurofibromatosis, pectus carinatum, and temporomandibular joint dysfunction: A case report

**DOI:** 10.1186/1754-9493-4-7

**Published:** 2010-05-03

**Authors:** Beyazit Zencirci

**Affiliations:** 1 MOSTAS Private Health Hospital, Department of Anesthesiology and Reanimation - Kahramanmaras, Turkey

## Abstract

**Background:**

Neurofibromatosis is a syndrome caused by the abnormal deposition of neural tissues of the nervous system, endocrine system, visceral structures, and skin. On the other hand, pectus carinatum and temporomandibular joint dysfunction are illnesses that adversly affect the respiratory system and cause additional problems in airway management.

**Case Presentation:**

Fifty-eight-year-old Turkish male patient had neurofibromatosis, pectus carinatum and temporomandibular joint dysfunction. The case was due to be operated on with the diagnosis of incarcerated umbilical hernia. Spinal anesthesia was successfully performed and the duration of the surgery was 1 hour. No postoperative complications were observed and he was discharged from the hospital on the 3rd post-operative day.

**Conclusion:**

The anesthetic management of patients with neurofibromatosis requires attention to all possible abnormalities and associated disturbances. Furthermore, the presence of pectus carinatum and temporomandibular joint dysfunction also increase the potential risks. The operation was successfully completed with spinal anesthesia that was carefully applied upon taking the required measures and considering all pathologies that may accompany the case and complications that may occur.

## Background

Von Recklinghausen disease, also known as neurofibromatosis 1 (NF1), is characterized by multiple cafe-au-lait spots in the skin, multiple peripheral nerve tumors, and a variety of other dysplastic abnormalities of the skin, nervous system, bones, endocrine organs, and blood vessels [1]. It is a genetic disease in humans, inherited by a gene located in chromosome 17, affecting one in 3000-4000 individuals [2].

Pectus carinatum (PC), also known as pigeon breast, is characterized by protrusion of the sternum and costal cartilages. This anomaly predominantly affects males at a ratio of 4:1 [3]. The typical PC deformity is first recognized during the early adolescent years and progressively becomes more severe until full skeletal maturity is reached, after which little change occurs throughout adulthood [4]. Some patients develop rigidity of the chest wall with decreased lung compliance, progressive emphysema, and increased frequency of respiratory tract infections [5].

Temporomandibular joint dysfunction (TMD) is a term used to describe a number of related disorders involving the temporomandibular joint (TMJ), masticatory muscles and associated structures [6]. The etiology of TMD is complicated and still largely unresolved. Malocclusion, psychogenic factors, and trauma, both chronic and acute, are often cited as possible causes or exacerbating events in patients vulnerable to TMD [7].

When evaluated from the point of general and regional anaesthesia; the case with particular and serious risks from various angles was that of an incarcerated umbilical hernia case.

## Case presentation

The 58 year-old Turkish male patient was 82 kg and 174 cm high. The case was due to be operated on with the diagnosis of incarcerated umbilical hernia.

**Figure 1:**
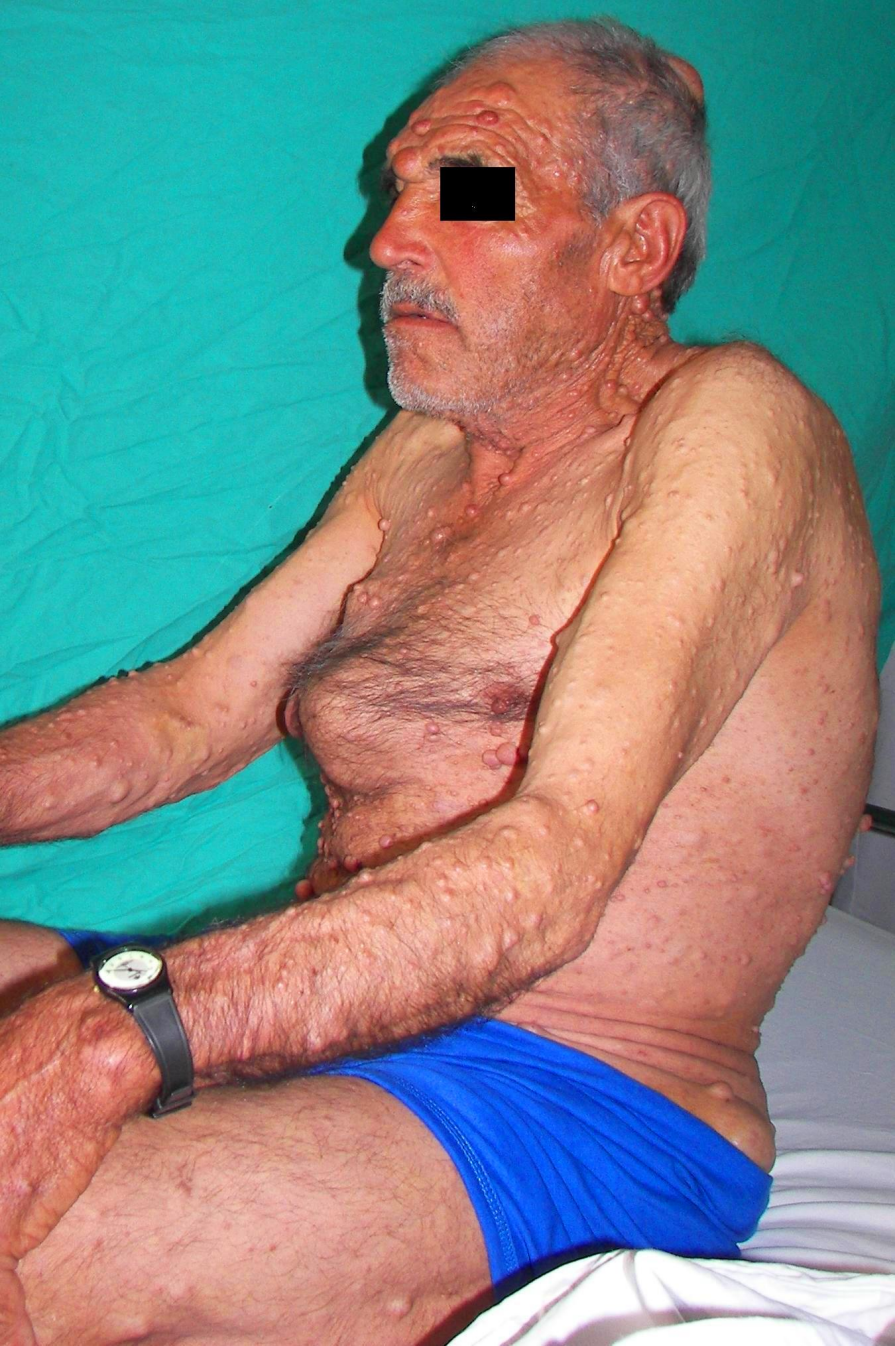
**Pectus carinatum and neurofibromatosis of the patient**.

**Figure 2:**
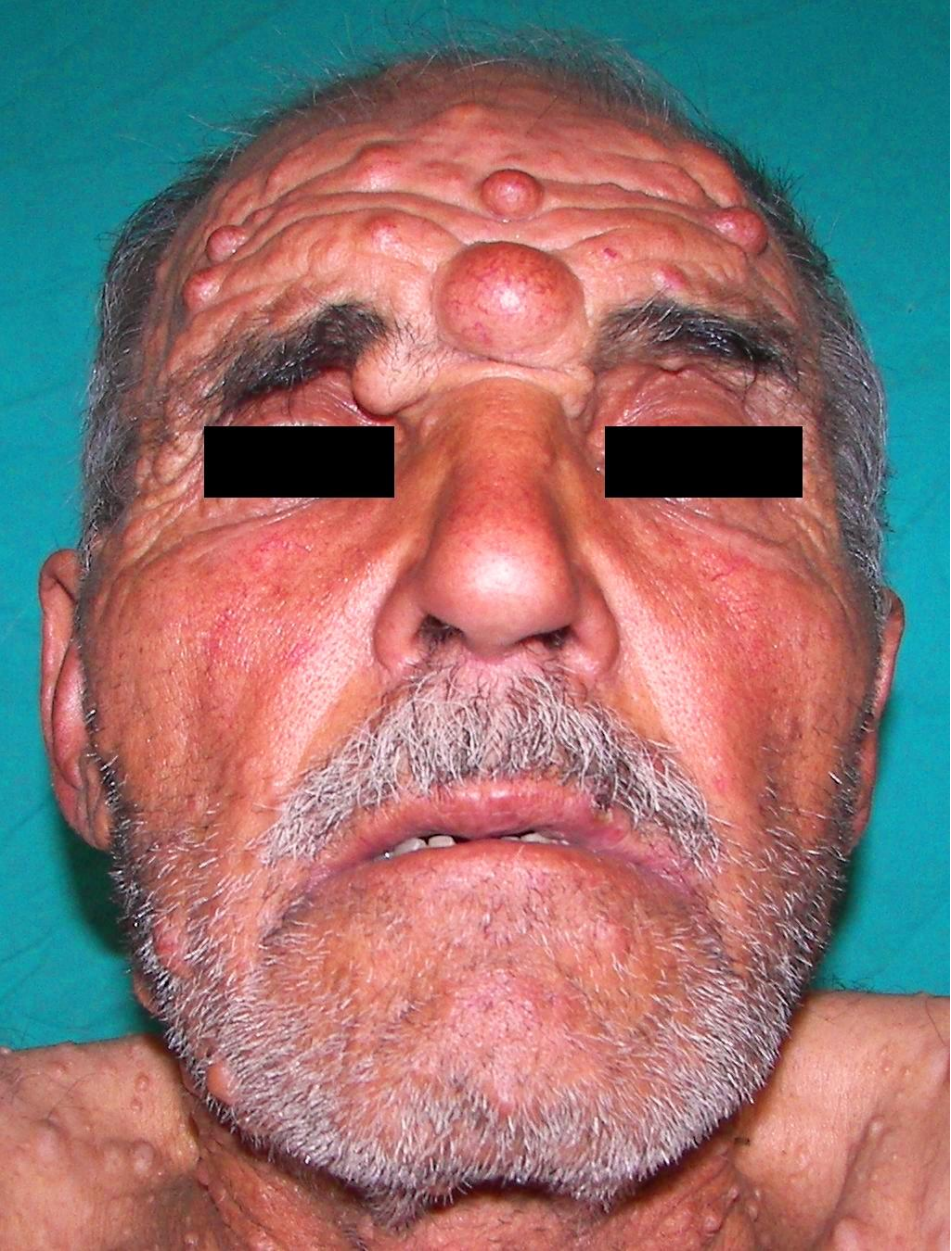
**Decreased mouth opening of the case (approximately 1.35 cm)**.

According to the physical examination, on the whole body of the case were various epidemic, indolent neurofibromatosis nodules of different sizes [Figure 1 and 2]. It was also understood during physical examination that the case had pectus carinatum deformity in the thorax zone [Figure 1].

On the other hand, according to patient's history, it was observed that the case had also had TMD (more apparent in the left temporomandibular joint) for about five years. Particularly, the case was complaining of sharp aches originating from the right pre-auricular zone and advancing towards the eye of the same side as well as periorbital and temporal areas. Another complaint of the case was the clicking sound that occured while chewing. Indeed, mouth-opening was seen to be a fingerbreadth (approximately 1.35 cm) during the physical examination, therefore mallampati score could not be assessed [Figure 2]. It was learned that the case had not accepted to have an operation due to his phobia although he was advised to have one for his TMD.

In preoperative laboratory assessment, apart from a slight leucocytosis (9.2 × 10^9^/L) and increased CRP (11.6 mg/dl), no other pathologies were observed. And in the electrocardiographic examination, an incomplete right branch block was found to be present.

When evaluated in the pre-operative visit, it was thought that regional anaesthesia application would be more appropriate for the patient and in order to exclude neurofibromatosis' central nervous system involvement, the case was applied a cranial and spinal MRI examination. No pathological findings were encountered in the MRI examination.

Considering the risks that may be caused by the available disturbances of the case (*especially, the possibility of difficult and even unsuccessful intubation and the risk of failure in providing a secure airway*), it was decided to apply spinal anesthesia on the case.

The patient, intravenously premedicated with midazolam 1.5 mg was monitored with electrocardiography (ECG), heart rate (HR), noninvasive blood pressure (NIBP), and pulse oximetry (SPO_2_) at the operating theatre. After administration of 500 mL lactated Ringer's solution; spinal anesthesia was successfully performed in the sitting position between lumbar 4 and 5 intervertebral spaces with a 27-gauge atraumatic spinal needle with 3 mL 0.5% hyperbaric bupivacaine. The level of the sensorial block was on thoracal 7, the operation was started in the 7th minute.

Besides, in consideration of unsuccessful anaesthesia or failure or the risk of a compulsion to turn back to general anaesthesia during the operation, an ear nose and throat specialist experienced in the subject of tracheostomy, a tracheostomy kit and sterile surgical tools were kept available in the operating theatre during the operation.

The patient tolerated the surgery very well without additional hypnotics. The duration of the uneventful surgery was 1 hour. All haemodynamic values were stable during surgery. The case that had been post-operatively observed in the intense care unit for one day was transferred to the surgical clinic without any complications observed. No postoperative complications were observed and he was discharged from the hospital on the 3rd post-operative day.

According to the monitoring carried out until the day of discharge, no complications such as headache, nausea, blood pressure change (*especially hypotension*) and neurological deficit that may develop secondary to spinal anesthesia were observed.

## Discussion

Neurofibromatosis type 1 (NF1) is an autosomal dominant disorder affected by NF1 gene. The NF1 gene encodes a protein named neurofibromin which has a role in tumour suppression. Inactivation of the gene leads to loss of function and subsequent development of many different types of tumours seen with the disease [8].

The choice of anesthetic technique in patients with NF1 requires careful systemic evaluation. Factors influencing airway management, respiratory and cardiovascular problems, central nervous system involvement, and vertebral anomalies make the choice between general and regional anesthesia more difficult.

Hypertension should be carefully examined in these cases. Because pheochromocytoma occurs in 0.1 to 5.7% of patients with NF1 [9]. Therefore after diagnosis of NF1, patients who have episodes of hypertension (*is the most consistent clinical sign*), sweating, headache and palpitation should be evaluated for pheochromocytoma. Urinary catecholamines and their metabolites may have increased. It would be suitable to start treatment with alpha-blocker (*phenoxybenzamine*) when preoperative hypertension is detected [10]. It should not be forgotten that an undefined or disregarded pheochromocytoma may lead to an intraoperative imminent and life-threatening hypertensive crisis in cases with neurofibromatosis. Consequently, although we had not detected hypertension during anamnesis and examination of the patient, the required plan for a probable hypertensive attack was made (*Administration dose and concentrations of a antihypertensive infusion agent was calculated and it was kept available to be administered at any time*).

Patient with NF1 have been reported to be either sensitive or resistant to succinylcholine. Furthermore, all reports indicate that response to nondepolarizing muscle relaxants is exaggerated [11,12]. Also prolonged apnea has been reported following both non-depolarizing and depolarizing muscle relaxants for unknown reasons [13].

Gliomas, meningiomas, hydrocephalus, and spina bifida have been found in NF1 patient. This may serve as a relative contraindication to spinal block anesthesia [14]. If it is perceived that neuraxial anesthesia is preferable because of the high risk of general anesthesia, spinal cord neurofibromas and intracranial hypertension need to be ruled out using CT or MRI [15]. According to the cranial and spinal MRI examination carried out for the purpose, no neurofibromatosis formations were observed in the case who had been decided to be applied spinal anaesthesia after the pre-operative evaluation. As the anamnesis, physical examination (including detailed *neurologic *examination) and cranial and spinal MRI findings of the patient supported the absence of these illnesses, we preferred to apply spinal anaesthesia.

Spinal block may be extremely difficult in a patient with NF1, as kyphoscoliosis or neurofibromas close to the needle puncture site may limit the safety of the procedure. Although some residual scars from his previous operation were present, they were not near the midline so we did not meet any technical difficulties. Epidural anaesthesia is often considered as contraindicated because neurofibromas may involve spinal cord and nerve roots [15].

Although rarely, pectus carinatum and NF1 may occur at the same time. Akçali et al. reports that they observed NF1 along with thorax deformity in one patient (rate 2.3%) out of 43 cases [16]. Pectus carinatum also occurs in association with scoliosis (15%), congenital heart disease, Marfan's syndrome, and other connective tissue disorders [17]. It often progresses in severity and becomes quite prominent during the active linear growth spurt of puberty, causing significant cosmetic and psychosocial concerns. In addition, patients frequently report symptoms such as chest pain and shortness of breath. On the other hand, asthmatic symptoms are more frequent in patients with carinatum than in those with excavatum [18].

The TMJ is a synovial joint that contains an articular disk which allows for hinge and sliding movements. This complex combination of movements allows for painless and efficient chewing, swallowing, and speaking [19]. TMJ may be affected by inflammatory, traumatic, infectious, congenital, developmental, and neoplastic diseases, as seen in other joints [20]. The most common symptom reported by patients with TMD is a pain centred in and around the pre-auricular region. Clicking or grating sounds on mandibular movement may also occur together with restricted mouth opening. Mandibular motion is usually limited, and attempts at active motion, such as chewing, talking, or yawning, increase the pain [6].

Adequate mouth opening is needed for normal feeding, speech and also blade insertion and rotation into the pharynx [21]. The normal range of motion of the TMJ should permit insertion of three fingers (approximately 5 cm) aligned vertically into the patient's mouth [22]. Limitation of mouth opening has long been the chief complaint for patients who have TMD.

Our case had sharp aches starting from the pre-auricular zone and sometimes advancing towards the eye of the same side as well as periorbital and temporal zones. Another complaint of the case was the clicking sound that occured while chewing. Indeed, mouth opening was seen to be a fingerbreadth (approximately 1.35 cm) [Figure 2].

In all NF1 patients with complicated airways, advanced planning as outlined in the American Society of Anesthesiologists' guidelines for the difficult airway management is advised [23]. A difficult airway cart and possibly equipment for emergency tracheostomy should be immediately available, depending on the severity of airway distortion. Throughout the operation, an ear nose and throat specialist experienced in the area of tracheostomy, a traceostomy kit and sterile surgical tools were kept available in the operating theatre for our case who, apart from the present NF1, already had high risk of intubation due to TMD.

Anesthetic experience in patients with neurofibromatosis is limited to few case reports in literature. The anesthetic challenges in these patients are many, and anesthetic management should be designed, based on the existing pathology and its severity. Careful pre-operative evaluation of the case, good preparation and selection and management of a close anaesthesia particularly avoided development of fatal airway and other complications.

## Conclusion

For our patient who required surgery, spinal anesthesia was the preferred method, as coexisting neurofibromatosis, pectus carinatum, and especially temporomandibular joint dysfunction might have worsened airway and respiratory status even the asymptomatic patient during general anesthesia. In conclusion, spinal anesthesia may be the safest practice in thoroughly evaluated patients with neurofibromatosis. Special caution must be taken to avoid neurological sequelae and uneventful anesthesia.

## Competing interests

The author declares that they have no competing interests.

## Consent

Written informed consent was obtained from the patient for the publication of this case report and accompanying images. A copy of the written consent is available for review by the Editor-in-Chief of this journal.

## References

[B1] Hirata D Nara H Inaba T von Recklinghausen disease in a patient with X-linked agammaglobulinemia Intern Med 2002 41 1039 1043 10.2169/internalmedicine.41.1039 12487187

[B2] Alkan A Sigirci A Kutlu R Neurofibromatosis type 1: diffusion weighted imaging findings of brain Eur J Radiol 2005 56 229 234 10.1016/j.ejrad.2005.05.008 15963674

[B3] Westphal FL Lima LC Lima Neto JC Prevalence of pectus carinatum and pectus excavatum in students in the city of Manaus, Brazil J Bras Pneumol 2009 3 221 226 10.1590/s1806-37132009000300005 19390719

[B4] Fonkalsrud EW Surgical correction of pectus carinatum: lessons learned from 260 patients J Pediatr Surg 2008 43 1235 1243 10.1016/j.jpedsurg.2008.02.007 18639675

[B5] Lee SY Lee SJ Jeon CW Effect of the compressive brace in pectus carinatum Eur J Cardiothorac Surg 2008 34 146 149 10.1016/j.ejcts.2008.04.012 18479931

[B6] Dimitroulis G Temporomandibular disorders: a clinical update Br Med J 1998 317 190 194 10.1136/bmj.317.7152.190 PMC1113540 9665905

[B7] Greene CS Marbach JJ Epidemiologic studies of mandibular dysfunction: a critical review J Prosthet Dent 1982 48 184 190 10.1016/0022-3913(82)90110-X 7050363

[B8] Reynolds RM Browning GGP Nawroz I Von Recklinghausen's neurofibromatosis: neurofibromatosis type 1 Lancet 2003 361 1552 1554 10.1016/S0140-6736(03)13166-2 12737880

[B9] Erem C Onder Ersöz H Ukinç K Hacihasanoglu A Alhan E Cobanoğlu U Koçak M Erdöl H Neurofibromatosis type 1 associated with pheochromocytoma: a case report and a review of the literature J Endocrinol Invest 2007 30 59 64 17318024 10.1007/BF03347397

[B10] Pacak K Preoperative management of the pheochromocytoma patient J Clin Endocrinol Metab 2007 92 4069 4079 10.1210/jc.2007-1720 17989126

[B11] Mitterschiffthaler G Maurhard U Huter O Prolonged action of vecuronium in neurofibromatosis von Recklinghausen's disease Anaesthesiol Reanim 1989 14 175 178 2568840

[B12] Naguib M Al-Rajeh SM Abdulatif M The response of a patient with von Recklinghausen's disease to succinylcholine and atracurium Middle East J Anesthesiol 1988 9 429 434 3143052

[B13] Manser J Abnormal responses in von Recklinighausen's disease Br J Anaesth 1970 42 183 10.1093/bja/42.2.183 4249575

[B14] Fisher MM Anaesthetic diffictulties in neurofibromatosis Anaesth 1975 30 648 10.1111/j.1365-2044.1975.tb00925.x 811128

[B15] Dounas M Mercier FJ Lhuissier C Epidural analgesia for labour in a parturient with neurofibromatosis Can J Anaesth 1995 42 420 422 10.1007/BF03015490 7614652

[B16] Akçali Y Ceyran H Hasdiraz L Chest wall deformities Acta Chir Hung 1999 38 1 3 10439083

[B17] Golladay ES Ziegler MM, Azizkhan RG, Weber TR Pectus carinatum and other deformities of the chest wall Operative Pediatric Surgery 2003 New York: McGraw-Hill 269 277

[B18] Fonkalsrud EW Beanes S Surgical management of pectus carinatum: 30 years' experience World J Surg 2001 25 898 903 10.1007/s00268-001-0048-x 11572031

[B19] Beuscher J Temporomandibular Joint Disorders Am Fam Physician 2007 76 1477 1482 18052012

[B20] Guralnick W Kaban LB Merrill RG Temporomandibular-joint afflictions N Engl J Med 1978 299 123 129 661872 10.1056/NEJM197807202990304

[B21] Kovacs G, Law JA Tracheal Intubation by Direct laryngoscopy Airway Management in Emergencies 2008 New York: McGraw-Hill 89

[B22] Kao SY Luo J Coronal approach for the replacement of the condylar head in bilateral temporomandibular joint ankylosis: report of three cases Chung-Hua-I-Hseueh-Tsa-Chih-Taipei 1999 62 388 394 10389298

[B23] Practice guidelines for management of the difficult airway An updated report by the American Society of Anesthesiologists Task Force on Management of the Difficult Airway Anesthesiology 2003 98 1269 1271 10.1097/00000542-200305000-00032 12717151

